# Effect of Metatarsophalangeal Joint Position on Maximal Voluntary Isometric Ankle Plantar‐Flexion Torque

**DOI:** 10.1111/sms.70390

**Published:** 2026-10-29

**Authors:** Hiroto Shiotani, Katsuki Takahashi, Kosei Taniai, Hayato Tsuru, Yasuo Kawakami

**Affiliations:** ^1^ Faculty of Sport Sciences, Waseda University Saitama Japan; ^2^ Research Organization of Science and Technology, Ritsumeikan University Kusatsu Shiga Japan; ^3^ Organization for Research Innovation, Doshisha University Kyoto Japan; ^4^ School of Sport Sciences, Waseda University Saitama Japan; ^5^ Human Performance Laboratory, Comprehensive Research Organization, Waseda University Tokyo Japan

**Keywords:** extrinsic and intrinsic foot muscles, plantar pressure, strength, toe flexor muscles, torque−angle relationship, windlass mechanism

## Abstract

The joint position at which maximal torque is produced indicates the optimal position for agonist muscles to function. It is well‐documented that the maximal voluntary isometric contraction (MVIC) torque of ankle plantar‐flexion varies with knee and ankle joint positions. However, metatarsophalangeal (MTP) joint position may also affect MVIC torque, reflecting force–length relationships of the toe flexor muscles. This study examined the effect of MTP joint position on MVIC torque of ankle plantar‐flexion. Sixteen young males performed MVICs of ankle plantar‐flexion with the MTP joint at 0°, 15°, 30°, and 45° of dorsiflexion. Ankle plantar‐flexion torque, plantar pressure distribution, and surface electromyography (sEMG) were recorded. MVIC torque was significantly greater at 15° than at 0°, 30°, and 45° (+11.0%–21.4%). Similarly, forces applied under the toes were significantly greater at 15° than at the other conditions (+13.4%–37.5%), whereas forces applied under the proximal foot and sEMG amplitudes of the major ankle plantar‐flexors remained comparable across MTP joint positions. At 45° MTP joint dorsiflexion, toe forces were 17.5% lower than at 30°, COP shifted 3–5 mm proximally, and sEMG amplitudes were 26.8% lower in abductor hallucis than at 30°, and 28.9% and 26.3% lower in flexor digitorum brevis than at 15° and 30°, respectively. These results demonstrate that MVIC torque of ankle plantar‐flexion varies with MTP joint position in an inverted U‐shaped manner and is maximized at 15° of MTP joint dorsiflexion, reflecting changes in toe flexor forces, proximodistal COP displacement, and sEMG amplitudes of the intrinsic foot muscles.

## 1 Introduction

In human locomotion (e.g., walking and running), the ankle plantar‐flexor muscles are major contributors to mechanical power production [1, 2, 3]. These muscles contribute substantially to body‐weight support, forward propulsion, and the initiation of leg swing [4]. In particular, the actions of the ankle plantar‐flexor muscle–tendon unit generate a burst of positive work during the push‐off phase [1, 3], which facilitates energy efficiency by redirecting the body during step‐to‐step transitions [5]. Therefore, the ankle plantar‐flexor muscles function as essential components of the physical capabilities required for both activities of daily living [6, 7] and athletic performance [2, 8, 9]. As such, maximal voluntary isometric contraction (MVIC) torque of ankle plantar‐flexion is widely assessed in clinical and athletic settings as a commonly used indicator of plantar‐flexor strength and function.

The joint position at which maximal torque is produced indicates the optimal position for the agonist muscles to function [10]. The triceps surae, a muscle group consisting of the gastrocnemius and soleus, spans the knee and/or ankle joints and inserts into the calcaneus via the Achilles tendon, and acts as the prime mover for ankle plantar‐flexion. It is well‐documented that MVIC torque varies with knee and ankle joint positions, and is maximized with the knee extended and ankle 0°–20° dorsiflexed position [11, 12, 13, 14, 15, 16], reflecting the force–length relationships of the triceps surae muscle. From a mechanical perspective, however, ankle plantar‐flexion torque reflects the external moment about the ankle joint generated by the ground/footplate reaction force acting on the foot. Therefore, forces acting through the forefoot (e.g., toe flexor forces) can contribute to the measured torque. Indeed, even after surgical excision of the Achilles tendon, the MVIC torque of the operated limb can reach up to 38% of that generated by the sound limb [17]. This residual plantar‐flexion torque is considered to be partly attributable to the extrinsic foot muscles, such as the flexor hallucis longus (FHL) and flexor digitorum longus (FDL). These muscles span the ankle and metatarsophalangeal (MTP) joints to insert into the distal phalanges, and act to generate ankle plantar‐flexion and toe flexion. Moreover, the human foot is not a rigid body, but rather a unique, arch‐shaped, deformable structure composed of numerous bones and articulations. During ankle plantar‐flexion, the intrinsic foot muscles, such as the abductor hallucis (ABH) and flexor digitorum brevis (FDB), act to resist foot arch deformation and generate toe flexion [18, 19, 20].

It has been reported that toe flexion torque (indicative of the toe flexor forces) varies with ankle and MTP joint positions and is maximized with the ankle 0°–20° dorsiflexed and MTP joint 25°–50° dorsiflexed positions [21, 22, 23]. These findings suggest that changes in MTP joint position may alter the force–length relationships of the toe flexor muscles (extrinsic and intrinsic foot muscles), thereby potentially affecting MVIC torque of ankle plantar‐flexion. A recent study attempted to examine the effect of MTP joint position on MVIC torque of ankle plantar‐flexion by comparing neutral and fully dorsiflexed MTP joint positions in combination with different knee and ankle joint positions [24]. However, no significant effect of MTP joint position on MVIC torque was observed. In their experimental setup, the toes were not in contact with the dynamometer footplate, which may have limited the contribution of toe flexor forces. This setup may not represent typical torque measurements, in which the toes remain in contact with the footplate or the terminal stance and push‐off phases of locomotion, during which the ground reaction force is mainly applied under the forefoot. Therefore, measuring plantar‐flexion torque under a condition in which the toes remain in contact with the support surface may be more reasonable for evaluating the effects of MTP joint position. Furthermore, full dorsiflexion of the MTP joint may induce shortening of the foot arch through the windlass mechanism [25, 26, 27]. This could partly offset the elongation of the intrinsic toe flexor muscles and shift the center of pressure (COP) proximally, thereby shortening the lever arm of the output ground reaction force. The reduced muscle elongation may attenuate the expected increase in the force‐generating capacity of the intrinsic foot muscles [21, 22, 23]. A shorter lever arm results in less torque for a given output force and is therefore mechanically unfavorable for ankle plantar‐flexion torque production [28, 29, 30]. Consequently, these mechanical changes may offset the potential increase in ankle plantar‐flexion torque resulting from increased toe flexor forces with MTP joint dorsiflexion. Thus, it is necessary to examine plantar‐flexion torque across intermediate MTP joint positions between neutral and fully dorsiflexed positions. Clarifying the optimal MTP joint position for MVIC torque would provide new insight into the functional role of the toe flexor muscles in ankle–foot plantar‐flexion.

Therefore, the purpose of this study was to investigate the effect of MTP joint position on MVIC torque of ankle plantar‐flexion. We hypothesized that MVIC torque varies with MTP joint position among 0°, 15°, 30°, and 45° of dorsiflexion, and is maximized with an intermediate joint position, thereby exhibiting an inverted U‐shaped pattern.

## 2 Materials and Methods

### 2.1 Participants

Sixteen healthy young males (age 21.7 ± 1.6 years, height 1.72 ± 0.06 m, body mass 63.7 ± 7.9 kg, and foot length 25.2 ± 1.0 cm; mean ± standard deviation [SD]) participated in this study. The sample size was determined by an a priori power analysis (G*Power v3.1; Heinrich Heine‐Universität Düsseldorf, Germany) for one‐way repeated measures analysis of variance (ANOVA); effect size *f* = 0.25, *α* = 0.05, 1 − *β* = 0.80, and a correlation among repeated measures *r* = 0.70, the required sample size was estimated to be *n* = 15. Females have relatively shorter foot lengths and lower arch heights than males [31]. To ensure compatibility with the experimental setup, we recruited male participants with similar foot lengths. Participants had no history of diagnosed foot deformities, such as pes planus, and showed no apparent foot abnormalities that would interfere with the measurements. All participants had no history of foot or lower limb injury or surgery within 12 months before the experiment. They were asked to refrain from any strenuous exercise for 24 h before the experiment. All participants completed a familiarization session before the main experiment, during which they practiced MVIC torque production under the same four MTP joint positions as those used in the main experiment. This session was conducted, on average, 5 days before the main experiment. This study was approved by the Human Research Ethics Committee of Waseda University (reference number: 2024‐387) and was carried out in accordance with the Declaration of Helsinki. Written informed consent was obtained from all participants before the experiments.

### 2.2 Data Collection

#### 2.2.1 Torque Measurements

Participants lay supine on the seat of a custom‐made myometer (VTF‐002, VINE, Japan) with their right knee fully extended, and the right thigh was strapped to the myometer seat. The right foot was firmly secured to the footplate with the ankle at a neutral position. The ankle joint center, approximated from the lateral and medial malleoli, was visually aligned with the rotation axis of the myometer. The MTP joint positions were set at 0°, 15°, 30°, and 45° of dorsiflexion based on nominal support‐surface angles. The 0° position was set using the flat footplate, whereas the other dorsiflexion angles were set using custom‐made attachments inclined at 15°, 30°, and 45° relative to the footplate (Figure 1). The medial aspect of the base of the first proximal phalanx was palpated and marked on the skin, and the lower edge of each attachment was aligned with this mark to standardize its position across conditions. The order of the MTP joint positions was randomized and counterbalanced across participants.

**FIGURE 1:**
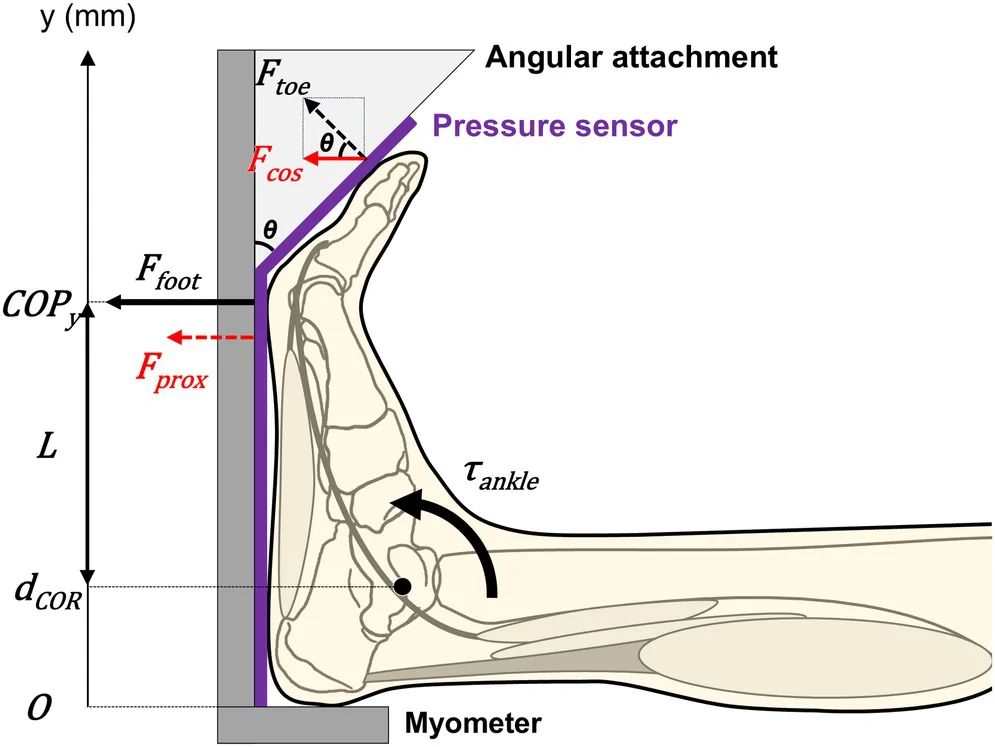
An illustration of experimental setup and parameters for plantar pressure‐based estimation of ankle plantar‐flexion torque.

Before testing, participants performed several submaximal ankle plantar‐flexion contractions (up to 80% of subjective maximal effort) as a warm‐up. Two MVICs of ankle plantar‐flexion were performed for each MTP joint position. Participants were instructed to push the footplate maximally with their forefoot, from the ball of the foot to the toes. A rest period of at least 1 min was provided between trials. The torque signal was amplified (DPM‐711B, Kyowa, Japan) and recorded at a sampling rate of 2000 Hz using a 16‐bit A/D converter (PowerLab 16s, ADInstruments, Australia) and stored on a personal computer running data acquisition/analysis software (LabChart ver.8, ADInstruments). Visual feedback of the torque signal and verbal encouragement were given to the participants to promote maximal effort for each trial. The MVIC torque was checked immediately after each trial, and if the difference in torque between the two trials exceeded 5%, an additional trial was performed. When three trials were performed, the two trials with the closest MVIC torque values were used for subsequent analyses.

#### 2.2.2 Plantar Pressure Measurements

Plantar pressure distributions underneath the foot during MVIC trials were measured using a plantar pressure measurement system (Pedar‐X, Novel GmbH, Germany). An insole‐type plantar pressure sensor (W.2640R, Novel GmbH) containing 99 capacitive sensors was firmly attached to the footplate. In the MTP joint dorsiflexed conditions, custom‐made attachments were inserted between the forefoot portion of the pressure sensor and the footplate, allowing the MTP joint to be secured at the required angle without preventing pressure measurement under the toes. The plantar pressure data were recorded at a sampling rate of 100 Hz and stored on a personal computer. To examine the possible changes in toe flexor forces during ankle plantar‐flexion, the forces applied under the toe and proximal foot (from the ball of the foot to the heel) regions were separately recorded. The boundary between the toe and proximal foot regions was determined before testing based on pressure distribution patterns obtained during several brief test procedures and was confirmed separately for each MTP joint position. These procedures included ankle plantar‐flexions with and without toe contact, toe flexions, and examiner‐applied pressure to the relaxed toes. Specifically, sensors showing foot contact and increased pressure during ankle plantar‐flexion without toe contact were assigned to the proximal foot region, and those responding during toe flexion and examiner‐applied pressure were assigned to the toe region. Before each trial, resting plantar pressure was excluded by zeroing the plantar pressure measurement system.

#### 2.2.3 Surface Electromyography

Surface electromyography (sEMG) signals during MVIC trials were recorded from the medial gastrocnemius (MG), soleus (SOL), tibialis anterior (TA), peroneus longus (PEL), FHL, ABH, and FDB using bipolar Ag/AgCl electrodes (Blue Sensor N‐00‐S; Ambu A/S, Denmark). The interelectrode distance was 20 mm for all muscles except FHL, for which it was 16 mm [32, 33]. The EMG signals were amplified (MEG‐6108; Nihon Kohden, Tokyo, Japan) and recorded at a sampling rate of 2000 Hz using the same A/D converter and computer software as for the torque signals to enable data synchronization. Before electrode placement, the muscle belly of each target muscle was confirmed using B‐mode ultrasonography (ProSound α7; Hitachi Aloka Medical, Japan), and the skin was shaved, abraded, and cleansed with alcohol. Electrodes were placed at 30% of the leg length (from the popliteal crease to the lateral malleolus) for MG, at 40% for TA and PEL, and at 40%–50% for SOL, adjusted individually based on the visualized superficial region of the muscle [34]. For FHL, electrodes were placed over the FHL muscle belly on the medial side of the leg between the distal end of the SOL muscle belly and the FHL muscle–tendon junction [33]. For ABH, electrodes were placed over the thickest region of the muscle belly on the medial aspect of the foot, approximately 1–2 cm posterior to the navicular bone. For FDB, electrodes were placed over the thickest region of the muscle belly on the plantar surface of the foot, beneath the navicular bone [35, 36].

### 2.3 Data Analysis

Data were processed using MATLAB 2024a (MathWorks, USA). The torque and plantar pressure data were low‐pass filtered at 10 Hz, and the sEMG data were band‐pass filtered between 20 and 450 Hz, using fourth‐order zero‐lag Butterworth filters. For each trial, the baseline passive torque recorded during the resting period before MVIC torque production was subtracted from the torque signal. The torque and plantar pressure data were synchronized based on the onset of ankle plantar‐flexion, defined as the time point at which the torque and total plantar force exceeded 2.0 Nm and 5.0 N, respectively. The MVIC torque was calculated for each trial, and the mean value of the two analyzed trials for each MTP joint position was used as the representative value.

From the plantar pressure data, the forces applied under the toe region (*F*_toe_) and proximal foot region (*F*_prox_), and center of pressure (COP) in the X (mediolateral; COP_*x*_) and Y (proximodistal; COP_*y*_) directions during each trial were obtained (Figure 1). *F*_toe_ and *F*_prox_ were extracted at the instant of MVIC torque, and COP coordinates were averaged over an eleven‐frame window centered on MVIC torque. The mean value of the two analyzed trials for each MTP joint position was used as the representative value. Furthermore, to examine the effect of MTP joint position‐dependent changes in toe flexor forces on MVIC torque, we estimated ankle plantar‐flexion torque from the plantar pressure data. First, the cosine (horizontal) component of *F*_toe_ at each MTP joint dorsiflexion angle (*θ*) was calculated as *F*_cos_, which acts perpendicular to the lever arm, contributing to ankle plantar‐flexion torque. The sine (vertical) component acts parallel to the lever arm and therefore does not contribute to the torque. The resultant horizontal force applied by the foot to the footplate (*F*_foot_) was calculated as the sum of *F*_cos_ and *F*_prox_:

$$F_{\cos}=F_{\mathrm{toe}}\cos\theta$$

$$F_{\text{foot}}=\left(F_{\cos}+F_{\text{prox}}\right)$$

The *y*‐coordinate origin of the plantar‐pressure sensor coordinate system (*O*; *y* = 0) corresponded to the posterior edge of the insole sensor. The distance *d*_COR_ was defined as the perpendicular distance from this reference point to the extended line of the rotation axis of the myometer. Because the insole sensor was firmly secured to the footplate and remained stationary throughout the experiment, *d*_COR_ remained constant. The *F*_foot_ was assumed to act at the calculated COP; accordingly, the lever arm (*L*) was calculated as the difference between COP_*y*_ and *d*_COR_. Then, ankle plantar‐flexion torque (*τ*_ankle_) was estimated from *F*_foot_ and *L*.

$$L=\left({\mathrm{COP}}_{y}-d_{\mathrm{COR}}\right)$$

$$\tau_{\text{ankle}}=F_{\text{foot}}\times L$$

For the sEMG data, the root mean square (RMS) values were calculated for each muscle over a 250‐ms window centered on the time point of peak torque. The mean value of sEMG‐RMS of the two analyzed trials was used as the representative value for each MTP joint position. With reference to the amplitude normalization matrix developed by consensus for experimental design in electromyography (CEDE) project, the sEMG‐RMS values were not normalized since the task of interest in the present study was MVIC torque production [37].

### 2.4 Statistical Analysis

The primary outcome measure was the ankle plantar‐flexion MVIC torque. Secondary outcome measures were plantar pressure and sEMG data. Normality of the data was assessed using the Shapiro–Wilk test. Sphericity was assessed using the Mauchly test, and the Greenhouse–Geisser correction was applied when the assumption was violated. A one‐way repeated measures ANOVA was performed to examine the effect of MTP joint position. If a significant main effect was found, post hoc pairwise comparisons were performed using paired *t*‐tests with Bonferroni correction. To test whether MVIC torque exhibited an inverted U‐shaped pattern across MTP joint positions, a within‐subject quadratic polynomial contrast was performed. As measures of effect size, partial eta‐squared ($\eta_{p}^{2}$) was calculated for the ANOVA and quadratic contrast, and Cohen's *d* for pairwise comparisons.

We assessed the correlation between the measured MVIC torque and the MVIC torque estimated from plantar pressure data using Pearson's product–moment correlation coefficient. To quantify estimation errors, the normalized root mean square error between the measured and estimated torque waveforms and the absolute percentage error of MVIC torques were calculated. To assess the agreement between measured and estimated MVIC torque, Bland–Altman analysis was performed separately for each MTP joint position. The differences (estimated minus measured MVIC torque) were plotted against their means. The bias was calculated as the mean of the differences, and the 95% limits of agreement (LoA) were defined as the mean difference ±1.96 SD of the differences. Proportional bias was assessed by linear regression of the differences between measured and estimated MVIC torque against their means. Statistical significance was set at *α* = 0.05.

## 3 Results

Figure 2 shows the changes in MVIC torque, *F*_toe_, *F*_cos_, and *F*_prox_ with MTP joint positions. A one‐way repeated measures ANOVA revealed a significant main effect of MTP joint position on maximal voluntary isometric ankle plantar‐flexion torque ($\eta_{p}^{2}$ = 0.506, *p* < 0.001), *F*_toe_ ($\eta_{p}^{2}$ = 0.472, *p* < 0.001), and *F*_cos_ ($\eta_{p}^{2}$ = 0.737, *p* < 0.001), respectively. No significant main effect of MTP joint position was observed for *F*_prox_ ($\eta_{p}^{2}$ = 0.040, *p* = 0.521). Post hoc pairwise comparisons showed that MVIC torque at 15° of MTP joint dorsiflexion was significantly greater than those at 0° (*d* = 1.276, *p* < 0.001), 30° (*d* = 1.030, *p* = 0.005), and 45° (*d* = 2.078, *p* < 0.001). A significant quadratic trend was observed in MVIC torque across MTP joint positions (*F*(1, 15) = 19.06, *p* < 0.001, $\eta_{p}^{2}$ = 0.560), consistent with the hypothesized inverted U‐shaped pattern. Similarly, *F*_toe_ and *F*_cos_ at 15° of MTP joint dorsiflexion were significantly greater than those at 0° (*d* = 1.276, *p* < 0.001, *d* = 1.023, *p* = 0.006), 30° (*d* = 0.936, *p* = 0.012, *d* = 1.855, *p* < 0.001), and 45° (*d* = 1.545, *p* < 0.001, *d* = 2.752, *p* < 0.001). *F*_toe_ and *F*_cos_ at 30° of MTP joint dorsiflexion were significantly greater than those at 45° (*d* = 0.954, *p* = 0.010, *d* = 1.687, *p* < 0.001).

**FIGURE 2:**
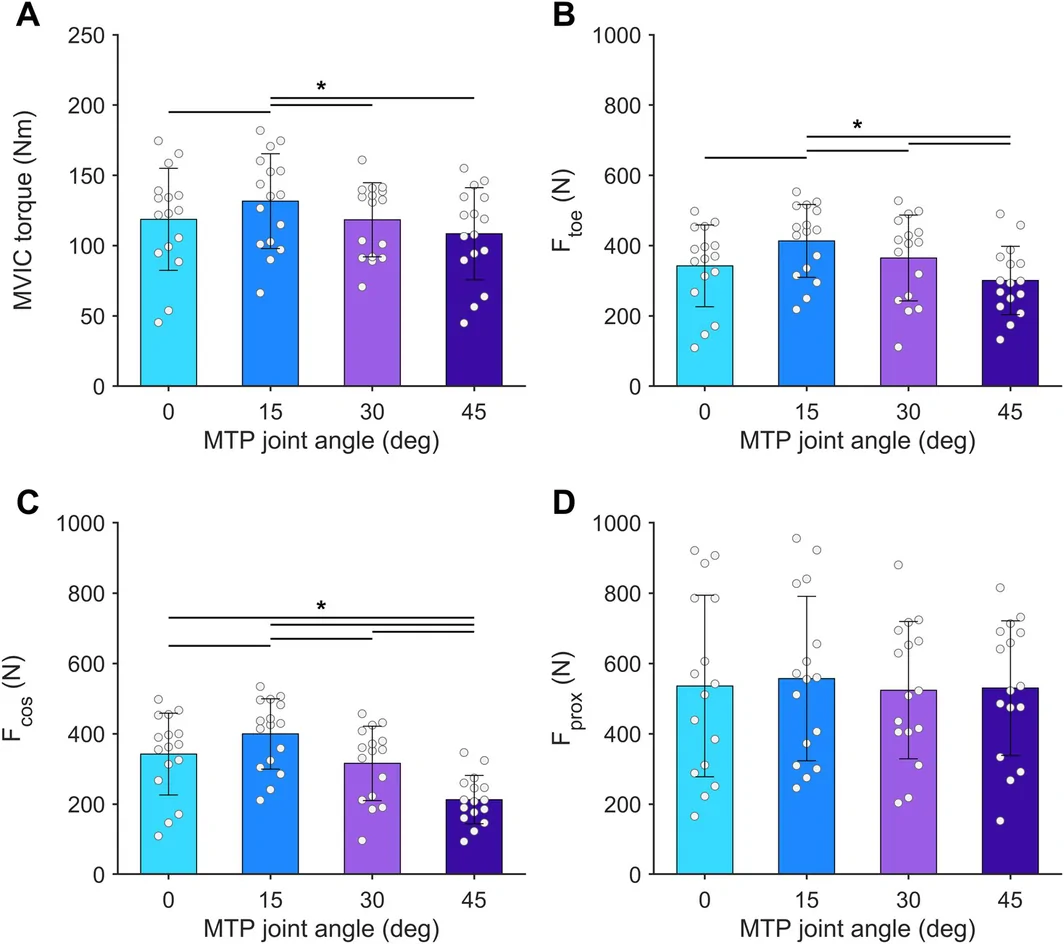
Data are shown as mean ± SD (*n* = 16). Changes in the MVIC torque of ankle plantar‐flexion (A) and *F*_toe_, *F*_cos_, and *F*_prox_ at the instant of MVIC torque (B–D) with MTP joint positions. A one‐way repeated‐measures ANOVA showed significant main effects of MTP joint position on MVIC torque, *F*_toe_, and *F*_cos_ (A–C; all *p* < 0.001), whereas no significant main effect was observed for *F*_prox_ (D; *p* = 0.521). * indicates significant differences between MTP joint positions in post hoc comparisons (*p* < 0.05).

There were significant positive correlations between measured and estimated MVIC torque at each MTP joint position (*r* = 0.919–0.978, all *p* < 0.001; Figure 3A). The intercept of each regression was not significantly different from zero (*p* = 0.348–0.938). The overall normalized root mean square error between the measured and estimated torque waveforms was 9.3% (angle‐specific means: 8.1%–11.7%), and the overall absolute percentage error between the measured and estimated MVIC torques was 12.2% (angle‐specific means: 9.2%–15.8%). The Bland–Altman analysis showed negative mean biases at all MTP joint positions (0°: −11.3 Nm [−26.9 to 4.3 Nm], 15°: −12.9 Nm [−35.1 to 9.3 Nm], 30°: −14.2 Nm [−35.4 to 6.9 Nm], 45°: −18.0 Nm [−41.7 to 5.7 Nm]; bias [95% LoA]; Figure 3B). No significant proportional bias was observed at any MTP joint position (*p* = 0.055–0.791).

**FIGURE 3:**
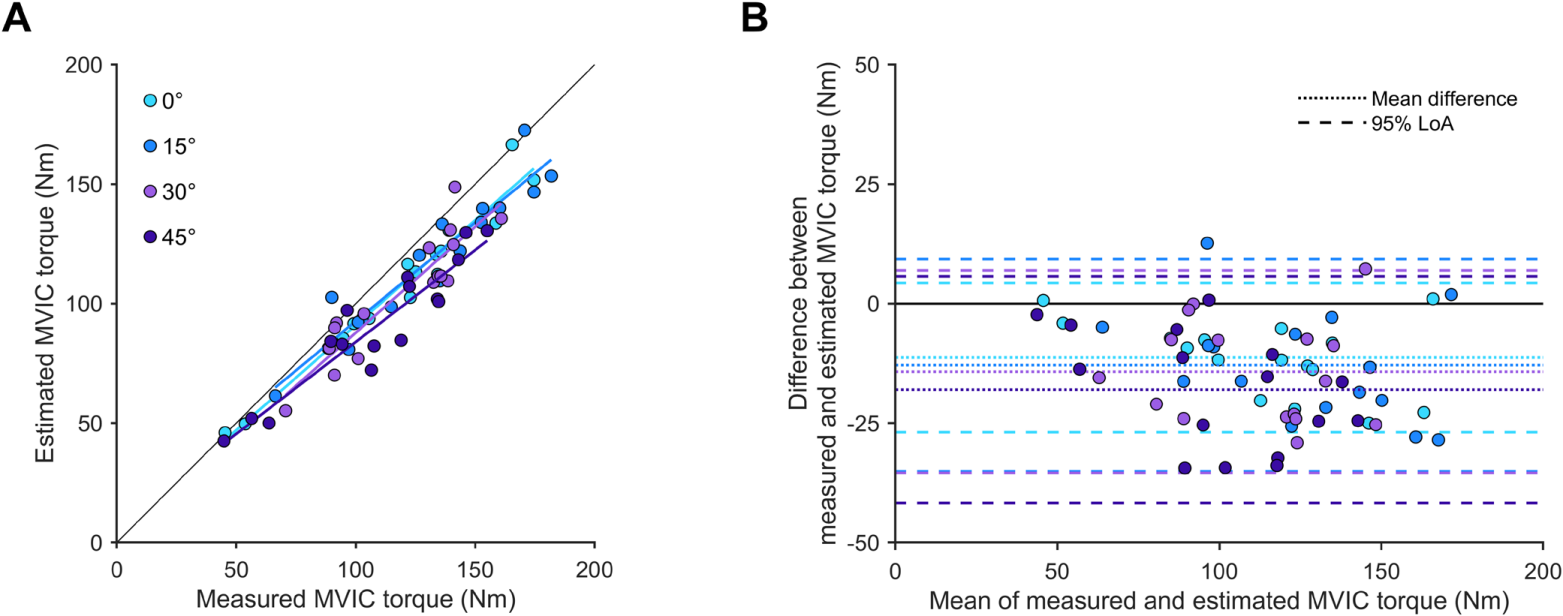
Correlation between measured MVIC torque and estimated MVIC torque based on plantar pressure data (A). The black line represents the identical line (*y* = *x*), and the colored lines represent the regression lines. Bland–Altman plots of the differences (estimated minus measured MVIC torque) against their means (B). The dotted and dashed lines represent the mean differences and 95% limits of agreement, respectively. In both panels, colors indicate MTP joint positions (*n* = 16 each).

Figure 4 shows the COP_*x*_ and COP_*y*_ at the instant of MVIC torque production for each MTP joint position. A one‐way repeated measures ANOVA revealed a significant main effect of MTP joint position on COP_*x*_ ($\eta_{p}^{2}$ = 0.216, *p* = 0.011) and COP_*y*_ ($\eta_{p}^{2}$ = 0.283, *p* = 0.002), respectively. Post hoc pairwise comparisons showed that COP_*x*_ was positioned significantly more laterally at 0° of MTP joint dorsiflexion than at 15° (*d* = 0.783, *p* = 0.041), and COP_*y*_ was positioned significantly more proximally at 45° of MTP joint dorsiflexion than at 15° and 30° (*d* = 0.842, *p* = 0.025 and *d* = 0.931, *p* = 0.012).

**FIGURE 4:**
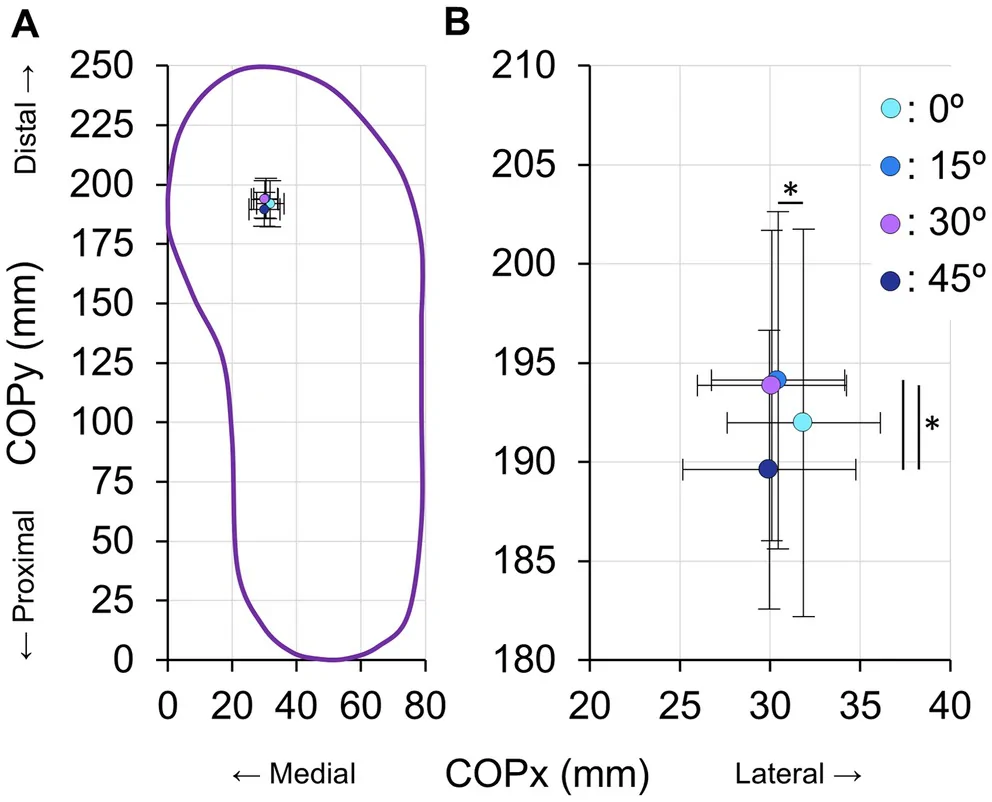
Data are shown as mean ± SD (*n* = 16). Overview (A) and enlarged view (B) of the COP positions at the instant of MVIC torque production for each MTP joint position. * indicates significant differences between MTP joint positions (*p* < 0.05).

A comparison of sEMG‐RMS values between MTP joint positions is shown in Table 1. A one‐way repeated measures ANOVA revealed a significant main effect of MTP joint position on the sEMG‐RMS of ABH ($\eta_{p}^{2}$ = 0.183, *p* = 0.041) and FDB ($\eta_{p}^{2}$ = 0.237, *p* = 0.028), whereas no significant main effects were observed for MG ($\eta_{p}^{2}$ = 0.056, *p* = 0.451), SOL ($\eta_{p}^{2}$ = 0.050, *p* = 0.505), TA ($\eta_{p}^{2}$ = 0.079, *p* = 0.286), PEL ($\eta_{p}^{2}$ = 0.151, *p* = 0.093), or FHL ($\eta_{p}^{2}$ = 0.086, *p* = 0.260). Post hoc pairwise comparisons showed that sEMG‐RMS of ABH was significantly lower at 45° of MTP joint dorsiflexion than at 30° (*d* = 0.862, *p* = 0.016) and that of FDB was significantly lower at 45° of MTP joint dorsiflexion than at 15° and 30° (*d* = 0.995, *p* = 0.007 and *d* = 0.897, *p* = 0.016).

**TABLE 1:** Comparison of sEMG‐RMS values between MTP joint positions.

sEMG‐RMS (mV)	MTP joint positions				ANOVA
	0°	15°	30°	45°	
MG	0.41 ± 0.23	0.43 ± 0.23	0.41 ± 0.23	0.40 ± 0.24	$\eta_{p}^{2}$ = 0.056, *p* = 0.451
SOL	0.21 ± 0.07	0.23 ± 0.07	0.21 ± 0.06	0.22 ± 0.09	$\eta_{p}^{2}$ = 0.050, *p* = 0.505
TA	0.11 ± 0.07	0.10 ± 0.07	0.09 ± 0.04	0.10 ± 0.06	$\eta_{p}^{2}$ = 0.079, *p* = 0.286
PEL	0.47 ± 0.25	0.47 ± 0.22	0.45 ± 0.21	0.40 ± 0.17	$\eta_{p}^{2}$ = 0.151, *p* = 0.093
FHL	0.19 ± 0.15	0.21 ± 0.15	0.23 ± 0.19	0.18 ± 0.16	$\eta_{p}^{2}$ = 0.086, *p* = 0.260
ABH	0.33 ± 0.16	0.36 ± 0.14	**0.36 ± 0.18** *	0.26 ± 0.14	$\eta_{p}^{2}$ = **0.183**, ***p* = 0.041**
FDB	0.31 ± 0.15	**0.29 ± 0.11** *	**0.28 ± 0.14** *	0.21 ± 0.10	$\eta_{p}^{2}$ = **0.237**, ***p* = 0.028**

*Note:* Data are shown as mean ± SD (*n* = 16). The bold values indicate significant main effects in the ANOVA and significant differences in pairwise comparisons (*p* < 0.05).

* Significant differences compared with 45° of MTP joint dorsiflexion (*p* < 0.05).

## 4 Discussion

The present study examined the effect of MTP joint position on MVIC torque of ankle plantar‐flexion. In support of our hypothesis, MVIC torque significantly varied with MTP joint position in an inverted U‐shaped manner and was maximized at 15° of MTP joint dorsiflexion. This finding partly supports previous evidence showing no significant difference in MVIC torque between the MTP joint at neutral and fully dorsiflexed positions [24], and further adds that an optimal MTP joint position for maximizing ankle plantar‐flexion torque exists at approximately 15° of dorsiflexion. Our results therefore demonstrate the effect of MTP joint position on ankle plantar‐flexion MVIC torque, extending the well‐established joint angle‐dependence at the knee and ankle [11, 12, 13, 14, 15, 16] to the MTP joint.

One of the unique aspects of the present study was the plantar pressure measurement during MVIC torque production, allowing us to quantify force distribution under the foot and to track changes in COP position. During MVIC torque production, *F*_cos_ was maximized at 15° of MTP joint dorsiflexion and decreased as the MTP joint was further dorsiflexed to 30° and 45°, showing a pattern similar to that of MVIC torque. In contrast, *F*_prox_ remained comparable across MTP joint positions. In the present study, the overall waveform and MVIC torque errors between the measured and estimated torques were 9.3% and 12.2%, respectively. These values were comparable to the errors of approximately 10%–15% or less reported in previous studies, in which plantar pressure‐based estimation of ankle plantar‐flexion torque was considered sufficiently accurate or clinically acceptable [38, 39]. Together with the strong correlations and absence of significant proportional bias, these findings suggest that the plantar pressure measurements sufficiently captured changes in the plantar‐force components contributing to ankle plantar‐flexion torque across MTP joint positions. Accordingly, the change in MVIC torque with MTP joint position was likely attributable to changes in the toe flexor forces, potentially reflecting the force–length relationships of the toe flexor muscles.

The optimal MTP joint position for maximizing MVIC torque of ankle plantar‐flexion in the present study slightly differed from the previously reported optimal position for toe flexion torque, approximately 25°–50° of dorsiflexion [21, 22, 23]. In the present study, *F*_toe_ was increased or maintained at dorsiflexed MTP joint positions compared with 0°, whereas *F*_cos_, the effective component contributing to ankle plantar‐flexion torque, significantly decreased beyond 15° up to 45° due to the cosine effect. If *F*_toe_ remained constant, *F*_cos_ would theoretically decrease by approximately 3.4% (cos15°), 13.4% (cos30°), and 29.3% (cos45°), respectively, compared with 0°. Thus, the increase in toe flexor forces with MTP joint dorsiflexion would be counterbalanced by the cosine effect. This trade‐off may explain why MVIC torque was maximized at 15° of MTP joint dorsiflexion, rather than at 30°–45°, where toe flexion torque has been reported to be maximized.

At 15° of MTP joint dorsiflexion, the COP_*x*_ was located approximately 1.5 mm more medially than at 0°, corresponding to the interval over which MVIC torque increased. A previous study examining toe flexion MVIC torque across MTP joint positions separately for the first and second–fifth MTP joints showed that the first MTP joint generated greater torque and exhibited a greater increase in torque with MTP joint dorsiflexion than the second–fifth MTP joints [22]. Therefore, the medial shift of the COP suggests that MTP joint dorsiflexion increased the relative contribution of the medial toes to force production.

Another important finding was that further MTP joint dorsiflexion beyond 15° was accompanied by a proximal shift of the COP. The COP_*y*_ at 45° of MTP joint dorsiflexion was located 3–5 mm more proximally than at 15° and 30°. The COP_*y*_ can vary with changes in plantar pressure distribution; however, at 45° MTP joint dorsiflexion, COP_*y*_ was also located 2.7 mm more proximally than at 0° despite no significant differences in *F*_toe_ or *F*_prox_ between these conditions. This suggests that the proximal shift of the COP was not solely attributable to reduced toe forces but also reflected changes in foot arch configuration, possibly arch shortening through the windlass mechanism. Such arch shortening may limit the elongation of the intrinsic foot muscles and shorten the lever arm, resulting in less torque for a given output force [28, 29, 30]. These changes may help explain why *F*_toe_ and ankle plantar‐flexion torque decreased at greater MTP joint dorsiflexion angles. In a simplified muscle–joint model, muscle length changes are generally described as a function of joint angles when a muscle spans one joint at which it produces movement [40, 41, 42]. Based on this model, extrinsic foot muscles such as FHL have been estimated to lengthen monotonically with MTP joint dorsiflexion [21], and the intrinsic foot muscles would be expected to lengthen similarly. However, in the multiarticular structure of the human foot, MTP joint dorsiflexion can induce shortening of the foot arch through the windlass mechanism [25, 26, 27]. Therefore, the length changes of the toe flexor muscles would be determined by the balance between muscle elongation due to MTP joint dorsiflexion and muscle shortening due to windlass‐related arch shortening. This may represent a unique example in which joint rotation does not directly translate into monotonic muscle lengthening.

The sEMG‐RMS of ABH and FDB decreased at 45° of MTP joint dorsiflexion, whereas no significant changes were observed in the leg muscles, including MG, SOL, TA, PEL, and FHL. To our knowledge, this is the first evidence that sEMG amplitudes of the intrinsic foot muscles vary with MTP joint position during MVIC torque production. However, in this study, possible angle‐ and muscle contraction‐dependent changes in non‐neural factors affecting sEMG amplitude, such as tissue geometry and electrode position relative to the underlying muscle fibers, were not assessed. Furthermore, given the nature of the maximal effort task, sEMG amplitudes were not normalized in the present study [37]. Thus, the present measurements cannot determine whether the lower sEMG amplitudes reflected changes in neural drive or non‐neural factors. Notably, the reductions in intrinsic foot muscle sEMG amplitudes from 30° to 45° were accompanied by a reduction in *F*_toe_ and a proximal shift of COP_*y*_. Previous tibial nerve anesthesia studies have demonstrated that blocking intrinsic foot muscle activation alters MTP joint mechanics and decreases ankle–foot torque/power production during walking, running, and jumping [20, 43]. These studies provide strong experimental evidence that the intrinsic foot muscles contribute to ankle–foot torque/power production. Thus, further studies are warranted to clarify MTP joint position‐dependent changes in the neuromechanical behavior of the intrinsic foot muscles, as well as the mechanisms underlying their contribution to ankle–foot torque/power production.

The present study has several limitations. First, the MTP joint positions were defined by the angles of the attachment surfaces relative to the footplate, and the actual anatomical MTP joint angles were not measured. In addition, because MTP joint position was standardized with reference to the hallux, the shorter lesser toes, particularly the lateral toes, may not have assumed the same dorsiflexion angles as the hallux. However, smaller MTP joint dorsiflexion toward the lateral toes has also been observed during push‐off in human locomotion [44], suggesting that such inter‐toe differences are not unique to the present experimental setup. Measurement of anatomical MTP joint angles may be useful in future studies requiring more detailed characterization of the MTP joint angle–torque relationship. Second, we examined only the isometric contraction mode with the ankle in the neutral position. Passive ankle joint angle changes can alter foot arch angle [26, 45]. Such foot arch deformation is accompanied by changes in intrinsic foot muscle length and thus consequently alters their force‐generating capacity. Although we considered testing combinations of ankle and MTP joint configurations in preliminary experiments, adding one ankle joint position would have required 8–12 additional maximal contractions, making it difficult to exclude the influence of fatigue. Future studies should examine the combined effects of ankle and MTP joint positions while carefully managing the influence of fatigue, and/or alternatively examine the isokinetic (concentric and eccentric) contraction modes to further clarify the contribution of toe flexor muscles to ankle plantar‐flexion and its underlying mechanisms. Third, interpretation of the sEMG data is limited by the potential effects of angle‐ and muscle contraction‐dependent non‐neural recording factors, the absence of amplitude normalization, and potential cross‐talk from adjacent muscles. Although ultrasonography was used to guide electrode placement, sEMG signals recorded over FDB may have included signals from deeper intrinsic muscles, such as the quadratus plantae [35]. However, because the quadratus plantae also acts to generate flexion of the second–fifth toes, potential crosstalk would not substantially alter the functional interpretation.

## 5 Conclusions

The present study demonstrated the effect of MTP joint position on MVIC torque of ankle plantar‐flexion. MVIC torque varied with MTP joint position in an inverted U‐shaped manner and was maximized at 15° of MTP joint dorsiflexion, reflecting changes in toe flexor forces. Further MTP joint dorsiflexion to 45° did not increase MVIC torque and was accompanied by reductions in toe flexor forces, a proximal shift of the COP, and reductions in sEMG amplitudes of the intrinsic foot muscles.

## 6 Perspectives

The present findings have practical implications for both plantar‐flexor strength assessment and human performance enhancement. First, MVIC torque during ankle plantar‐flexion was substantially influenced by MTP joint position, indicating that toe position should be controlled or at least carefully reported during plantar‐flexor strength testing. This is particularly relevant for measurements using handheld dynamometers in which only the ball of the foot contacts the device [46]. Uncontrolled MTP joint configuration may lead to misinterpretation of plantar‐flexor strength and function. Second, MVIC torque was maximized at approximately 15° of MTP joint dorsiflexion, suggesting that an optimal forefoot configuration may exist for ankle–foot torque/power production. This concept may be relevant to sports performance and footwear design, as modern running shoes often incorporate curved carbon‐fiber plates [47, 48, 49]. Notably, greater carbon‐fiber plate curvature has been shown to reduce ankle push‐off torque relative to a flat plate [47]. Although the specific curvature magnitudes and MTP joint angles were not reported, this result may be partly consistent with the present finding that further MTP joint dorsiflexion reduced MVIC torque. Future studies should examine the in‐shoe foot joint configurations induced by footwear designs and clarify whether optimizing MTP joint configuration can enhance ankle–foot torque/power production and athletic performance.

## Funding

This work was supported by TOBE MAKI Foundation (23‐JC‐001).

## Conflicts of Interest

The authors declare no conflicts of interest.

## Data Availability

The data that support the findings of this study are available from the corresponding author upon reasonable request.
